# Supplementary figures and images for: Functional and Taxonomic Effects of Organic Amendments on the Restoration of Semiarid Quarry Soils
Source: mSystems. 2021 Nov 23;6(6):e00752-21. doi: 10.1128/mSystems.00752-21 (PMC8609970; doi:10.1128/mSystems.00752-21)

Most Prevalent Pyhla (>1% summed across all samples)

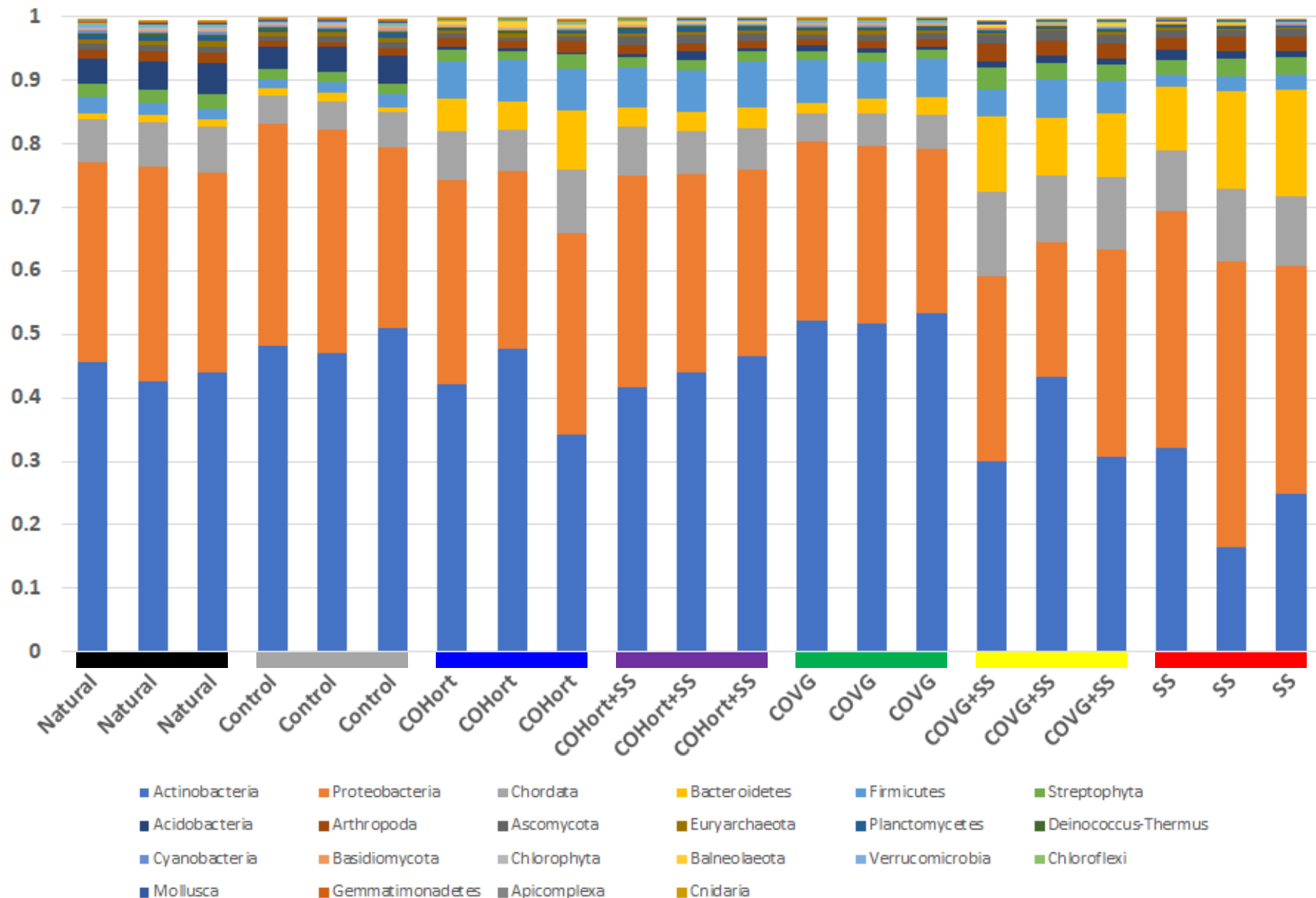

Supplement: FIG S1 [file msystems.00752-21-sf001.pdf]

Most Prevalent Genera (>10% summed across all samples)

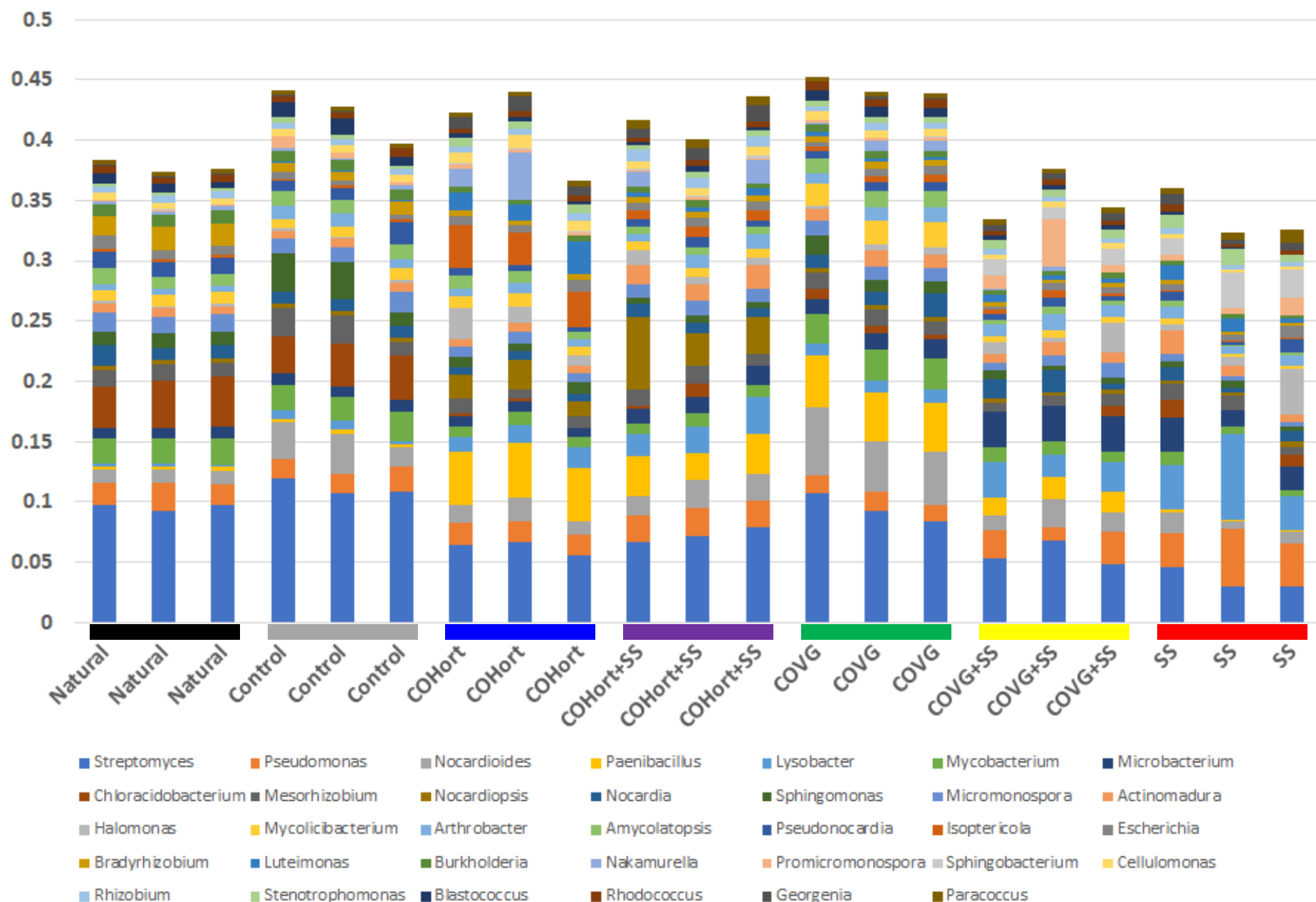

Supplement: FIG S2 [file msystems.00752-21-sf002.pdf]

**A**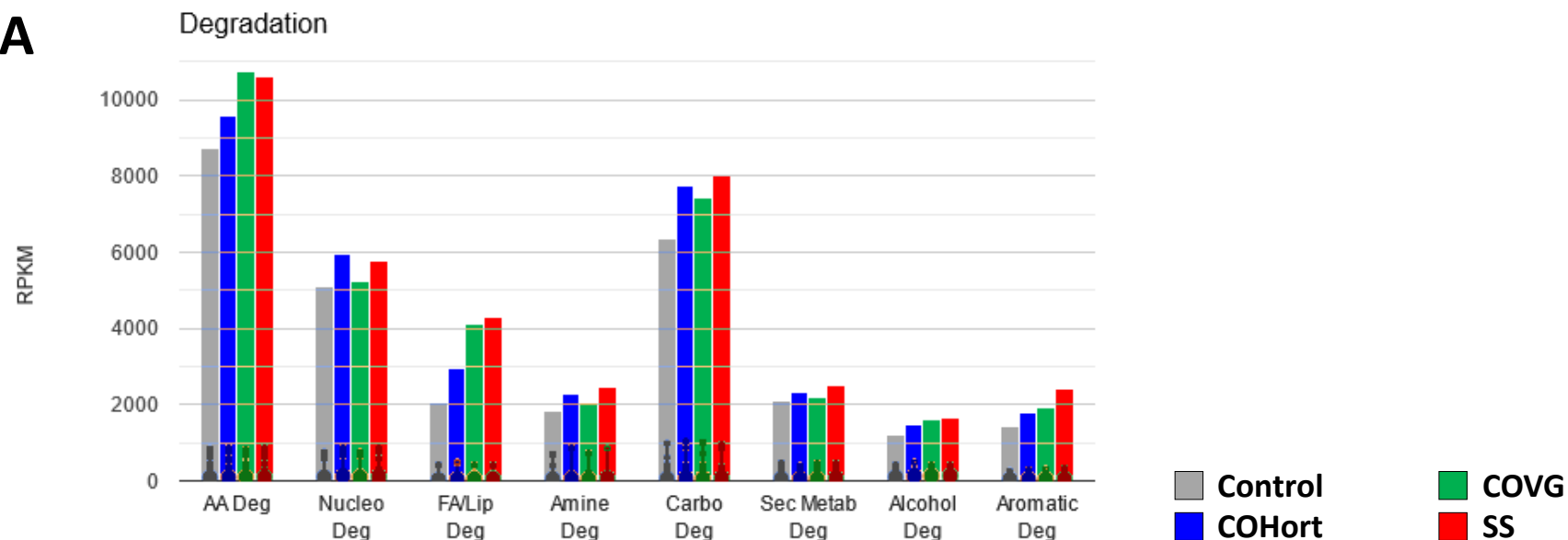**B**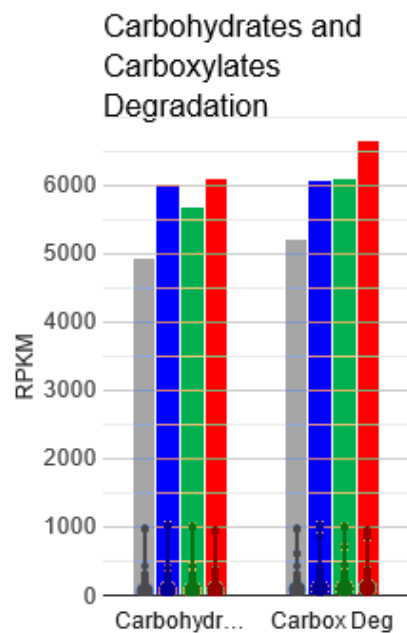**C**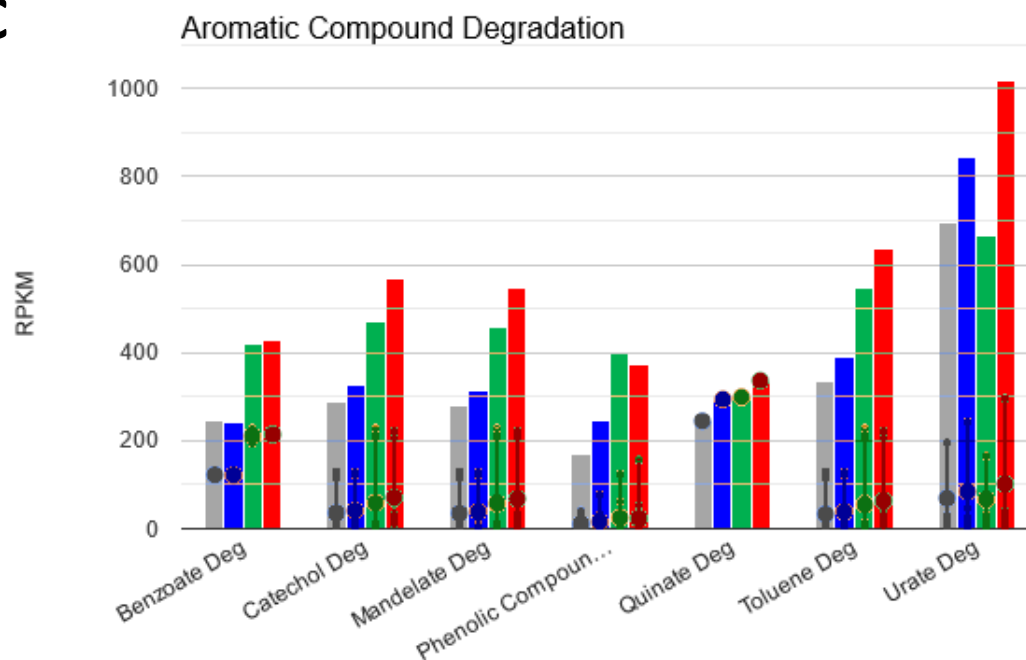

Supplement: FIG S4 [file msystems.00752-21-sf004.pdf]

**A**

### Biosynthesis

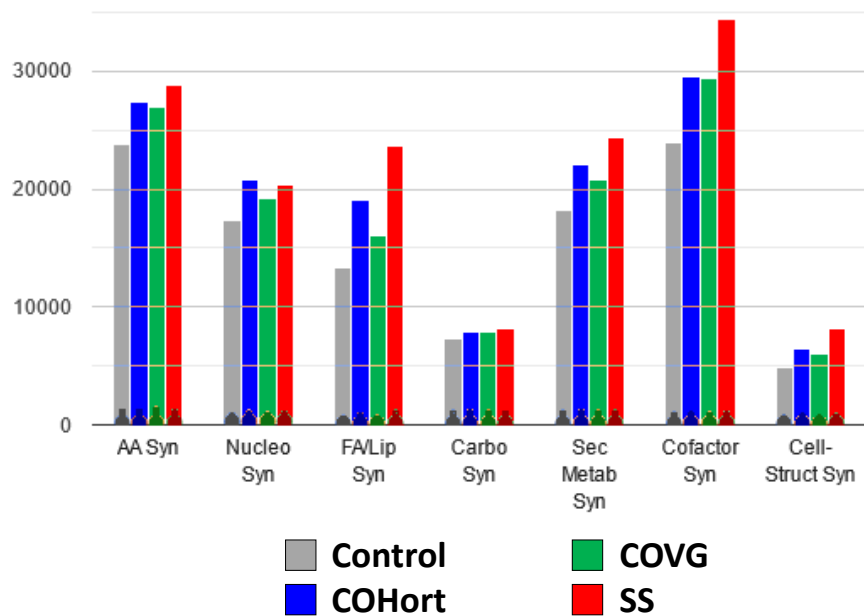

**B**

### Carbohydrate Biosynthesis

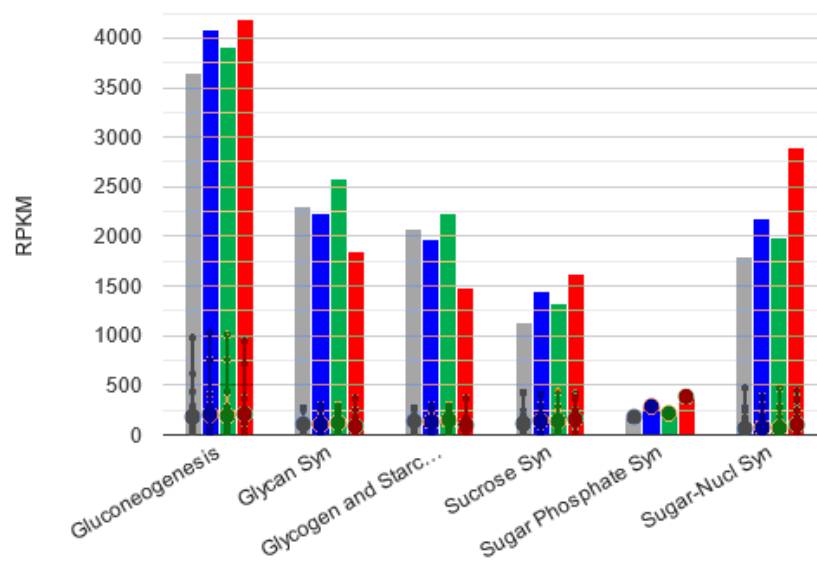

Supplement: FIG S3 [file msystems.00752-21-sf003.pdf]

**A****Energy**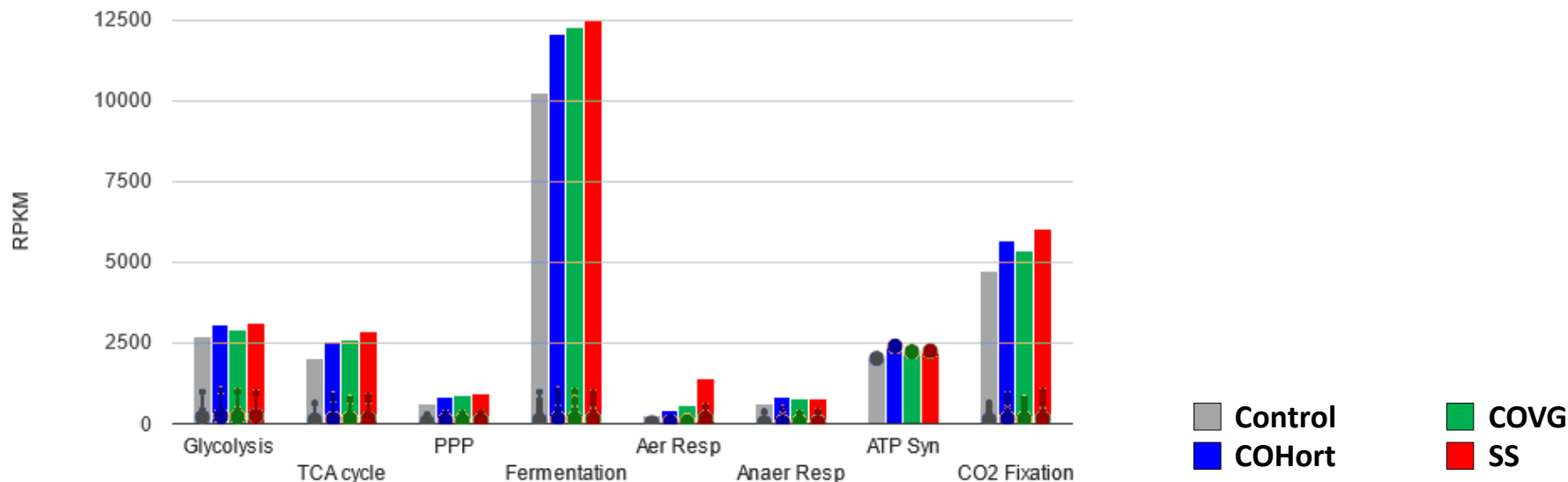**B****Other Pathways**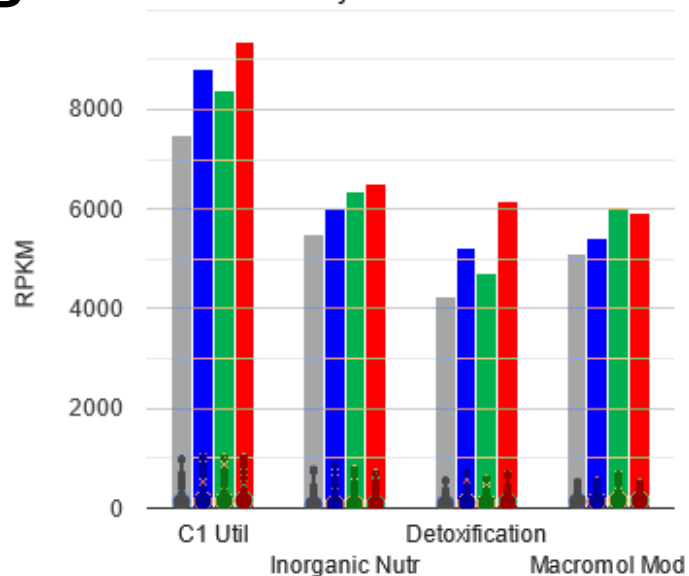**C****Inorganic Nutrient Metabolism**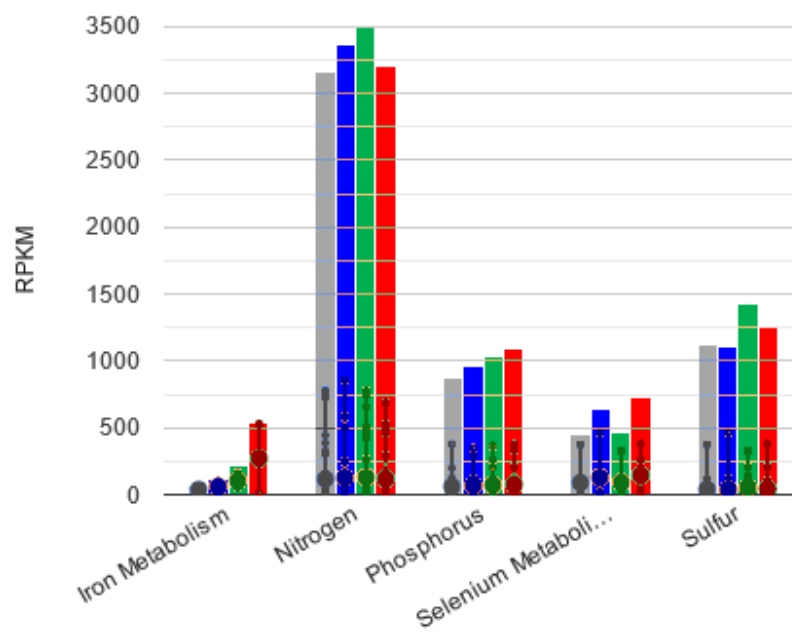

Supplement: FIG S5 [file msystems.00752-21-sf005.pdf]

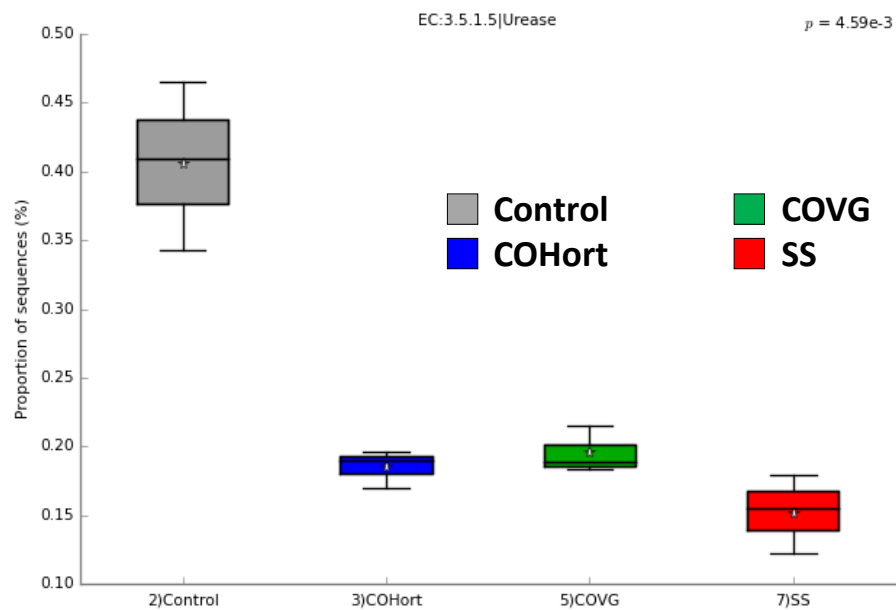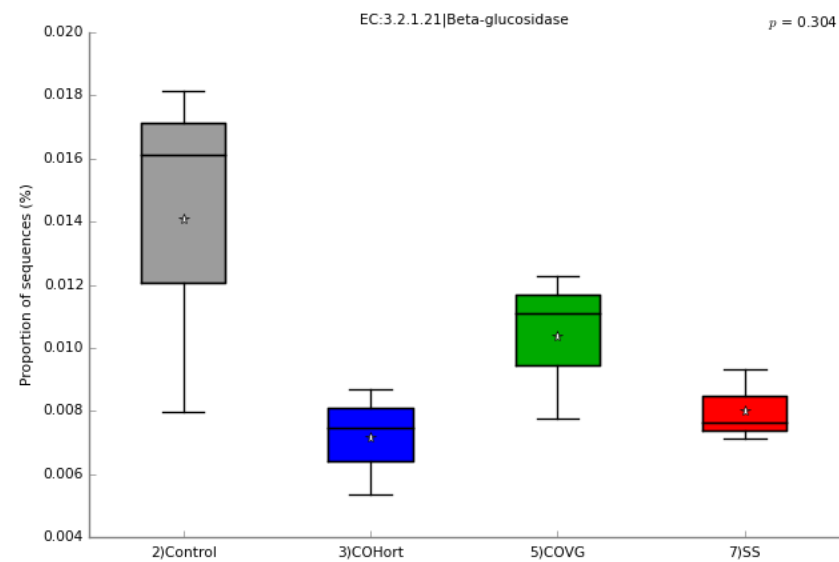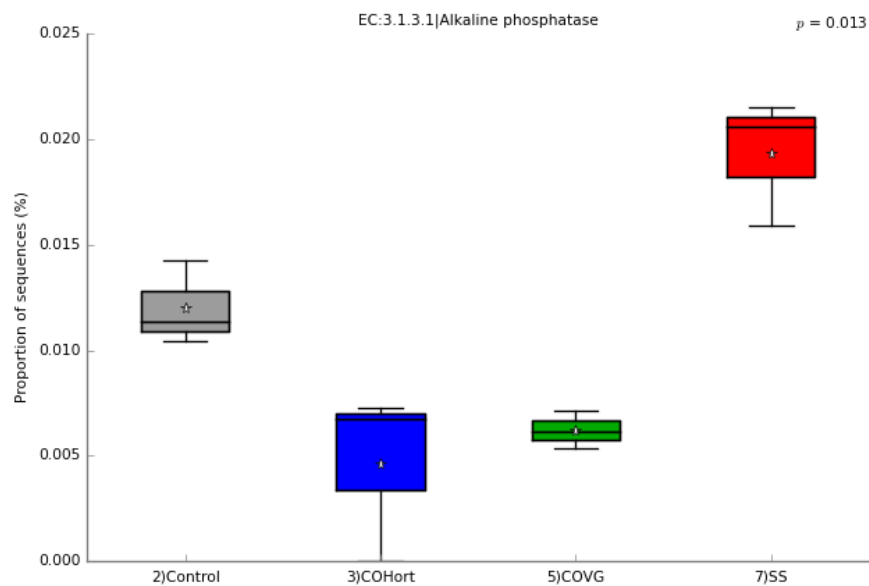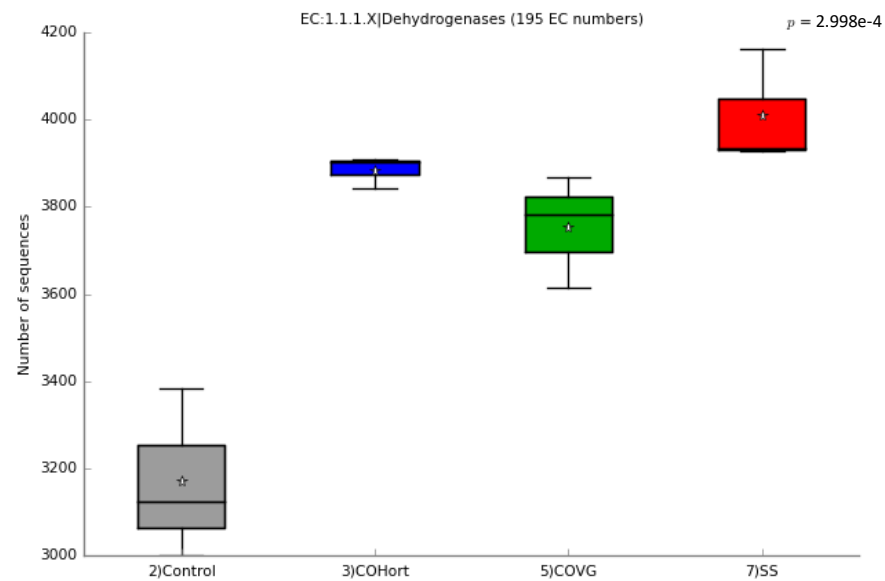

Supplement: FIG S6 [file msystems.00752-21-sf006.pdf]
